# MEASURING INTERGENERATIONAL AMBIVALENCE TOWARD OLDER ADULTS IN THE FAMILY CONTEXT: A SCOPING REVIEW

**DOI:** 10.1093/geroni/igae098.2730

**Published:** 2024-12-31

**Authors:** Athena Chung Yin Chan, Sophia Lee, Shuwen Li, Jacquelyn Duckett, Rodlescia Sneed

**Affiliations:** Texas Tech University, Lubbock, Texas, United States; Texas Tech University, Lubbock, Texas, United States; Texas Tech University, Lubbock, Texas, United States; Wayne State University, Detroit, Michigan, United States; Wayne State University, Detroit, Michigan, United States

## Abstract

Informal family caregivers may frequently feel ambivalent toward taking care of older family members. Ambivalence is the simultaneously held conflicting emotions, cognitions and/or behavioral tendencies due to countervailing expectations about how individual should act within the cultural norms. Most existing measurements directly or indirectly measuring caregiving or intergenerational ambivalence were developed from the West. Less is known how ambivalence is understood across distinctive sociocultural context globally. This scoping review examines the dimensions of existing instruments in measuring caregiver ambivalence toward older adults. Peer-reviewed articles were identified in five electronic databases. A keyword search was performed associated with ambivalence, caregiver or family, and measure development. Ten instruments were included. These instruments used different terms to represent caregiver ambivalence, not limited to intergenerational ambivalence and filial maturity. Existing measure items primarily capture the simultaneously held conflicting emotions toward older family members but limited to the domains of behaviors or cognitions. Moreover, existing measures are not inclusive of family caregiving to capture ethnic minorities who provide support to older family members motivated by filial responsibility beliefs. The scoping review uncovers the gaps between conceptual understanding and measurement of caregiver ambivalence toward older adults in the family. Findings will inform the direction of future research and the development of a culturally relevant measure that captures the intergenerational ambivalence experienced by ethnic minority who provide family support to older adults. The review will likely inform practice policy, and research in caregiving in a culturally sensitive manner.

